# Pain Management in Patient‐Facing Pharmacy Settings in Jordan: Pharmacists’ Knowledge, Attitudes, and Practices

**DOI:** 10.1155/prm/9198210

**Published:** 2026-08-28

**Authors:** Walid Al-Qerem, Judith Eberhardt, Lujain Al-Sa’di, Sarah Abu Hour, Dunia Basem, Lama Sawaftah

**Affiliations:** ^1^ Department of Pharmacy, Faculty of Pharmacy, Al-Zaytoonah University of Jordan, Amman 1173, Jordan, zuj.edu.jo; ^2^ Department of Psychology, School of Social Sciences, Humanities and Law, Teesside University, Middlesbrough TS1 3BX, UK, tees.ac.uk

**Keywords:** chronic pain, community pharmacists, Jordan, knowledge attitudes and practices, pain management, patient counseling

## Abstract

**Background:**

Community pharmacists are well placed to support pain management, yet evidence on their knowledge, attitudes, and practices remains limited in some settings, including Jordan.

**Objectives:**

This study examined pharmacists’ knowledge, attitudes, practices, perceived barriers, and training needs related to pain management among pharmacists working in patient‐facing pharmacy settings in Jordan.

**Methods:**

A cross‐sectional survey was conducted among 408 licensed pharmacists working in community and hospital pharmacies in Jordan. A structured, self‐administered questionnaire assessed pain management knowledge, counseling practices, perceived competence, regulatory knowledge, barriers to practice, and training preferences. Descriptive statistics and regression analyses were performed.

**Results:**

Pharmacists demonstrated generally good foundational knowledge and high levels of engagement in patient counseling. However, gaps were identified in pediatric pain perception, analgesic safety, and the integration of nonpharmacological approaches. Fear of dispensing opioids and uncertainty around controlled substance regulations were the commonly reported barriers. Counseling frequency was positively associated with both objectively assessed knowledge and self‐rated knowledge, with perceived competence showing a stronger association.

**Conclusions:**

Pharmacists in patient‐facing pharmacy practice in Jordan play an active role in pain management but face gaps in applied clinical understanding and regulatory confidence. Practice‐focused, guideline‐based training may help strengthen pharmacists’ contribution to chronic pain care.

## 1. Introduction

Pain, regardless of its underlying cause, severity, and whether it is physical or psychological in nature [1], represents a major medical and public health issue. It affects approximately 20% of the global population, with a further 10% of adults diagnosed with chronic pain for the first time each year [2]. A systematic review reported that between 13% and 51% of individuals living in resource‐limited settings experience chronic pain [3]. Beyond prevalence, pain has a substantial negative impact across multiple domains of life, including physical functioning, mental health, and financial wellbeing. Compared with the general population, individuals with chronic pain are more than twice as likely to experience reduced work capacity and up to four times more likely to report mood disorders [4, 5]. Effective pain management therefore requires an approach that combines pharmacological and nonpharmacological strategies [6].

In many cases, community pharmacists serve as the first point of contact for patients seeking health‐related advice due to their availability and affordability [7]. Pain is the most common reason for visits to community pharmacies [8]. As a result, pharmacists play a key role in pain management through developing patient‐focused treatment plans, monitoring treatment progress and potential drug interactions, and providing patient education [9]. Pharmacist‐led interventions and patient counseling have been shown to improve pain outcomes, anxiety, depression, and physical functioning while also reducing adverse drug reactions [10]. Community pharmacists, as medicine experts and patient educators, play an important role in improving clinical outcomes for people experiencing pain [11].

tHowever, despite extensive research and clinical guidance, many patients continue to experience avoidable pain in both inpatient and outpatient settings [12]. Gaps in healthcare professionals’ knowledge, along with limited coordination between providers, remain key barriers to effective pain management [13]. Evidence from the Middle East indicates that community pharmacists may be insufficiently prepared for this role. Studies conducted in Saudi Arabia and the United Arab Emirates have reported limitations in community pharmacists’ knowledge, attitudes, and behaviors related to pain management, highlighting the need for targeted education and training [14, 15].

While the role of community pharmacists in pain management is well recognized, there remains a limited evidence base examining their knowledge, attitudes, and practices (KAP), particularly in specific national contexts. In Jordan, such evidence is notably sparse.

The pharmacy workforce in Jordan is predominantly distributed across community and hospital pharmacy settings, with community pharmacies representing the largest sector and serving as highly accessible healthcare points for the public. Community pharmacists play a central role in medication dispensing, patient counseling, and supporting self‐care, often functioning as the first point of contact within the healthcare system [16]. Similarly, hospital outpatient pharmacists perform roles comparable to those of community pharmacists, including dispensing medications and counseling ambulatory patients. Understanding pharmacists’ knowledge, attitudes, and practices in this context is therefore important for identifying opportunities to strengthen their contribution to pain management within the Jordanian healthcare system.

The healthcare system in Jordan has regulatory characteristics that may shape pharmacists’ participation in pain management [17]. Community pharmacies are widely available and often serve as a primary point of care due to their walk‐in access, lower costs, and extended hours [18]. Therefore, strict regulations governing controlled substances, including opioid analgesics, may affect pharmacists’ confidence and engagement in opioid‐related pain management [19]. Additionally, variability in access to multidisciplinary pain services and limited structured continuing education may influence pharmacists’ preparedness and practice. These factors underscore the importance of examining pharmacists’ knowledge, attitudes, and practices within the Jordanian context [20]. This study therefore aimed to examine the factors influencing pain management decisions among pharmacists working in community and hospital outpatient pharmacies in Jordan, with a focus on their knowledge, attitudes, and practices.

## 2. Methods

### 2.1. Study Design and Population

This study employed a cross‐sectional survey design to assess pharmacists’ knowledge, counseling practices, perceived competence, and perceived barriers related to pain management. Data were collected between April and September 2025. Eligible participants were licensed pharmacists practicing in community and hospital outpatient pharmacies who consented to take part in the study. Hospital outpatient pharmacies were included because they provide patient‐facing dispensing and counseling services to ambulatory patients. A structured, self‐administered electronic questionnaire, accompanied by an informed consent form, was distributed both in person and via social media platforms. The survey was distributed using a combination of in‐person and online methods. Face‐to‐face recruitment took place in community and hospital outpatient pharmacies across Jordan, where the research team provided tablets for participants to complete the survey on‐site or shared a survey link for completion on their own devices. The online survey was distributed through professional pharmacist groups on social media platforms. Participation was voluntary, and electronic informed consent was obtained before participants accessed the survey. Participants were informed of the study purpose, their right to decline participation or withdraw before submission, and that their responses would be used for research purposes only. No information that could identify individual participants was collected, and all responses were anonymous. Data were stored securely and accessed only by the research team. To enhance geographic representativeness, a clustered sampling approach was applied by region and sex. Jordan was divided into three geographic clusters (Northern, Central, and Southern regions), and regional targets were informed by the national distribution of registered community pharmacists (Central: 2423 [67.6%], Northern: 855 [23.8%], Southern: 307 [8.5%]) [21], which was used as a proxy for the distribution of community pharmacies. Pharmacists were recruited within each cluster through on‐site visits to community and hospital outpatient pharmacies and through online dissemination. The achieved regional distribution of participants (Central: 305 [74.8%], Northern: 69 [16.9%], Southern: 34 [8.3%]) was broadly consistent with national workforce patterns, and significant differences were found between the sample distribution and the distribution of pharmacies overall; full national versus sample counts are provided in Appendix Table A1. Moreover, we ensured that the majority of participants were female to reflect the gender distribution of pharmacists in Jordan, where females constitute approximately two‐thirds of the workforce [22, 23]. The study was conducted in accordance with the ethical principles of the Declaration of Helsinki, and ethical approval was obtained from the Institutional Review Board of Al‐Zaytoonah University of Jordan (Ref. No. 11/13/2024–2025).

### 2.2. Study Instrument

Data were collected using a structured, self‐administered electronic questionnaire titled The Role of Jordanian Pharmacists in Pain Management. The instrument was adapted from a previously validated questionnaire developed by Mujtaba et al. [6] that assessed community pharmacists’ knowledge and professional practices related to pain management. The questionnaire consisted of four main sections: (1) sociodemographic and professional characteristics; (2) knowledge, skills, and competence in the use of analgesics for pain management; (3) self‐reported confidence and perceived competence in pain management; and (4) perceived barriers to pharmacists’ role in pain management.

### 2.3. Tool Validation

The content validity of the questionnaire was assessed by an expert panel comprising two pharmacists and two clinical pharmacists. The questionnaire was originally developed in English and subsequently translated using a forward–backward translation approach to ensure linguistic and conceptual equivalence. Two independent translators translated the questionnaire into Arabic, producing two initial versions. These versions were reviewed and reconciled by the translators and the research team to produce a single preliminary Arabic draft. The draft was then back‐translated into English by two additional independent translators and compared with the original questionnaire. The original version, back‐translated version, and Arabic draft were reviewed collectively by the research team to produce the final Arabic version. This process followed the Brislin translation principle to ensure cultural relevance and conceptual accuracy [24].

Face validity was assessed through a pilot study conducted with 30 pharmacists prior to the main data collection. Participants were recruited from community pharmacies and hospital outpatient pharmacies across different regions of Jordan, ensuring representation of the main practice settings included in the study. All participants received an explanation of the study purpose and provided informed consent after eligibility was confirmed. Participants were asked to complete the questionnaire and comment on item wording, clarity, and ease of understanding. Feedback was reviewed by the research team and used to refine item phrasing and improve clarity. Data from the pilot study were used solely for validation purposes and were excluded from the final statistical analysis.

### 2.4. Sample Size Calculation

The minimum required sample size for this cross‐sectional survey was estimated using the single‐population proportion formula for prevalence studies, *n* = *Z* ^2^ *p*(1 − *p*)/*d* ^2^[26]. A conservative expected proportion of 0.50 was assumed for pharmacists with adequate knowledge and counseling practices related to pain management. A 95% confidence level (*Z* = 1.96) and a margin of error of 5% (*d* = 0.05) were applied. This yielded a minimum required sample size of 384 pharmacists. To account for potential nonresponse and incomplete questionnaires, the target sample size was increased to 400. The final analysis included 408 fully completed questionnaires. The questionnaire was designed with mandatory response fields to prevent missing data; therefore, no incomplete responses were included in the analysis.

### 2.5. Statistical Analysis

Statistical analyses were conducted using SPSS version 26. Descriptive statistics were used to summarize participant characteristics and study variables. Data normality was assessed using the Kolmogorov–Smirnov test and indicated non‐normal distributions; therefore, nonparametric approaches were applied. Continuous variables are presented as medians with interquartile ranges (IQRs), and categorical variables are reported as frequencies and percentages.

Pharmacists’ knowledge, skills, and competence related to pain management were assessed using 10 items measured on a 5‐point Likert scale ranging from strongly disagree (1) to strongly agree (5), yielding a maximum possible score of 50. Negatively worded items were reverse‐coded prior to score calculation. Patient counseling practices were assessed using four items measured on a 4‐point Likert scale ranging from never (1) to most of the time (4), resulting in a maximum possible score of 16, with higher scores indicating better knowledge and more frequent counseling behaviors.

Associations between pharmacist characteristics and outcome measures were examined using regression analyses. Internal consistency of the questionnaire was assessed using Cronbach’s alpha, which was 0.60 for the knowledge score and 0.70 for the patient counseling practice score. Quantile regression was used to identify factors associated with pharmacists’ knowledge and patient counseling practice scores, given their non‐normal distributions. Ordinal logistic regression was performed to examine predictors of pharmacists’ self‐reported knowledge of regulations governing controlled substances. Multicollinearity was assessed using variance inflation factors (VIFs), which were below 3 across all models, indicating no significant multicollinearity. Statistical significance was set at *p* < 0.05.

## 3. Results

Table 1 presents the sociodemographic and professional characteristics of the participating pharmacists. A total of 408 pharmacists were included, of whom 81.9% were female, with a median age of 26 years (IQR: 24–30). Most participants held a Bachelor’s degree in Pharmacy (60%), followed by PharmD degrees (16.7%) and diploma qualifications (12.5%), while 10.8% reported postgraduate education. Nearly half of the sample had 1–4 years of professional experience, and 29.9% were recent graduates, reflecting a predominantly early‐career workforce.

**TABLE 1:** Sociodemographic and professional characteristics of the study participants.

		Count
Age		26 (24–30)

Gender	Female	334 (81.9%)
	Male	74 (18.1%)

Educational level	Diploma	51 (12.5%)
	Bachelor’s degree in Pharmacy	245 (60%)
	Doctor of Pharmacy (Pharm.D)	68 (16.7%)
	Postgraduate	44 (10.8%)

Years of experience	Recently graduated (< 1 year)	122 (29.9%)
	1–4 years	184 (45.1%)
	5–10 years	58 (14.2%)
	> 10 years	44 (10.8%)

Workplace	Community pharmacy chain	96 (23.5%)
	Independent community pharmacy	233 (57.1%)
	Governmental hospital	41 (10%)
	Private hospital	38 (9.3%)

Work region	Southern region	34 (8.3%)
	Northern region	69 (16.9%)
	Central region	305 (74.8%)

Have you undergone training specifically related to pain management?	No	259 (63.5%)
	Yes	149 (36.5%)

Where did you receive training in pain management?	No training received	214 (52.5%)
	During my school education	34 (8.3%)
	At the pharmacy	108 (26.5%)
	Other	52 (12.7%)

Did you receive your pharmacy education in Jordan?	No	17 (4.2%)
	Yes	391 (95.8%)

Most pharmacists worked in independent community pharmacies (57.1%), followed by chain pharmacies (23.5%), government hospitals (10.0%), and private hospitals (9.3%). The majority practiced in the central region (74.8%). More than half of the participants reported no prior training in pain management, while 36.5% reported having received training. Among those who had received training, the most common sources were the pharmacy workplace (26.5%) and pharmacy school education (8.3%). Nearly all participants (95.8%) had completed their pharmacy education in Jordan.

Pharmacists’ knowledge, skills, and competence in pain management are summarized in Table 2. Most respondents demonstrated good understanding of several core concepts. More than 80% agreed that analgesic selection should be guided by pain intensity and that cognitive behavioral therapy (CBT) is effective for chronic pain management. Similarly, a high proportion recognized the cardiovascular risks associated with nonsteroidal anti‐inflammatory drugs (NSAIDs) and supported the use of combination analgesic therapy with different mechanisms of action to improve pain control while minimizing adverse effects. Most participants (85.8%) agreed or strongly agreed that opioid doses should be titrated according to patient response.

**TABLE 2:** Pharmacists’ knowledge, skills, and competence in pain management.

	Strongly disagree	Disagree	Neutral	Agree	Strongly agree
The “WHO analgesic ladder” for children does not include weak opioids	7 (1.7%)	19 (4.7%)	52 (12.7%)	170 (41.7%)	160 (39.2%)
Do infants feel less pain than adults in similar situations?	21 (5.1%)	101 (24.8%)	122 (29.9%)	131 (32.1%)	33 (8.1%)
Treatment of chronic pain only with analgesic and adjuvant painkillers is effective in most patients	98 (24%)	18 (4.4%)	96 (23.5%)	148 (36.3%)	48 (11.8%)
Use of over‐the‐counter Paracetamol is contraindicated in asthma and pneumonia	97 (23.8%)	134 (32.8%)	69 (16.9%)	69 (16.9%)	39 (9.6%)
Choice of recommended pain reliever should depend on pain intensity	3 (0.7%)	21 (5.1%)	42 (10.3%)	165 (40.4%)	177 (43.4%)
Cognitive behavioral therapy (CBT) is very effective in the treatment of chronic pain and should be used as early as possible in the treatment plan for most patients with chronic pain	1 (0.2%)	7 (1.7%)	63 (15.4%)	199 (48.8%)	138 (33.8%)
NSAIDs (non‐steroidal anti‐inflammatory drugs) may increase the risk of cardiovascular disease, including heart attack, stroke, heart failure, and atrial fibrillation	7 (1.7%)	26 (6.4%)	68 (16.7%)	197 (48.3%)	110 (27%)
Antidepressants usually help relieve symptoms and improve function in patients with chronic pain	6 (1.5%)	25 (6.1%)	81 (19.9%)	220 (53.9%)	76 (18.6%)
Combining analgesics that act by different mechanisms (e.g., combining an NSAID with an opioid) may result in better pain control with fewer side effects than using a single analgesic agent.	6 (1.5%)	35 (8.6%)	92 (22.5%)	188 (46.1%)	87 (21.3%)
After an initial dose of an opioid analgesic is given, subsequent doses should be adjusted in line with the individual patient’s response	2 (0.5%)	7 (1.7%)	49 (12%)	243 (59.6%)	107 (26.2%)

*Note:* Items 1–4 were reverse‐coded.

Several knowledge gaps were also identified. A notable proportion of respondents incorrectly believed that infants experience less pain than adults, and more than one‐quarter agreed that paracetamol is contraindicated in patients with asthma or pneumonia. Responses were mixed regarding whether chronic pain can be effectively managed using analgesics and adjuvant medications alone. In contrast, most participants recognized that antidepressants can improve symptoms and functional outcomes in patients with chronic pain.

When asked to evaluate the adequacy of their pharmacy education related to narcotics and pain management, as well as their knowledge of laws governing controlled substances, nearly half of respondents rated their educational preparation as moderate, while approximately one‐quarter rated it as high or very high, suggesting an overall positive perception of their academic training (Table 3). The median knowledge score was 37 (IQR: 34–39) out of a maximum possible score of 50. Similarly, most pharmacists reported moderate levels of knowledge regarding laws and regulations related to controlled substances.

**TABLE 3:** Perceived quality of pharmacy education in pain management and knowledge of laws governing controlled substances.

		Count (%)
How satisfactory was your pharmacy education regarding narcotics and pain management?	very low	32 (7.8%)
	Low	69 (16.9%)
	Moderate	201 (49.3%)
	High	84 (20.6%)
	very high	22 (5.4%)

Your knowledge of laws regarding controlled substances	very low	0 (0%)
	Low	50 (12.3%)
	Moderate	195 (47.8%)
	High	126 (30.9%)
	very high	37 (9.1%)

When pharmacists were asked to rate their overall knowledge of pain management on a five‐point scale, more than half rated themselves at 4 or 5, while only a small proportion rated themselves at 1 or 2. Similar response patterns were observed for self‐reported familiarity with over‐the‐counter (OTC) pain management guidelines and confidence in advising on nonpharmacological approaches (Table 4). Most pharmacists reported recommending paracetamol rather than ibuprofen. The majority indicated a need for additional training in pain management, and nearly all respondents agreed that clinical guidelines are both useful and important for practice (Table 5).

**TABLE 4:** Self‐reported familiarity with pain management guidelines, confidence in advising on nonpharmacological strategies, and overall pain management knowledge, *n* (%).

	Very low	Low	Moderate	High	Very high
Are you familiar with guidelines on OTC painkillers?	18 (4.4%)	28 (6.9%)	119 (29.2%)	144 (35.3%)	99 (24.3%)
How confident do you feel when giving advice on alternative nonpharmacological pain management strategies?	18 (4.4%)	51 (12.5%)	143 (35%)	111 (27.2%)	85 (20.8%)
How would you rate your knowledge of pain management on a scale from 1 to 5?	10 (2.5%)	31 (7.6%)	152 (37.3%)	163 (40%)	52 (12.7%)

**TABLE 5:** Overall assessment of knowledge, skills, and training needs in analgesic‐based pain management, *n* (%).

	Strongly disagree	Disagree	Neutral	Agree	Strongly agree
My general preference is to recommend paracetamol over ibuprofen	4 (1%)	17 (4.2%)	69 (16.9%)	189 (46.3%)	129 (31.6%)
I need some training regarding pain management	14 (3.4%)	43 (10.5%)	95 (23.3%)	188 (46.1%)	68 (16.7%)
Clinical guidelines are useful when dealing with pain	2 (0.5%)	3 (0.7%)	38 (9.3%)	228 (55.9%)	137 (33.6%)

Counseling behaviors related to pain management are presented in Table 6. Pharmacists reported frequently counseling patients on medication side effects, potential drug interactions, therapeutic alternatives, and nonpharmacological options. Few respondents reported rarely or never engaging in these counseling activities, suggesting high levels of patient interaction and counseling engagement. The median counseling score was 14 (IQR: 12–16) out of a maximum possible score of 16.

**TABLE 6:** Patient counseling practices in pain management provided by pharmacists.

	Never	Rarely	Sometimes	Most of the times
To what extent do you provide education about side effects?	2 (0.5%)	25 (6.1%)	147 (36%)	234 (57.4%)
To what extent do you provide education about drug interactions?	3 (0.7%)	47 (11.5%)	171 (41.9%)	187 (45.8%)
To what extent do you provide education about alternatives?	1 (0.2%)	32 (7.8%)	116 (28.4%)	259 (63.5%)
To what extent do you provide education about nonpharmacological strategies?	6 (1.5%)	41 (10%)	163 (40%)	198 (48.5%)

**FIGURE 1:**
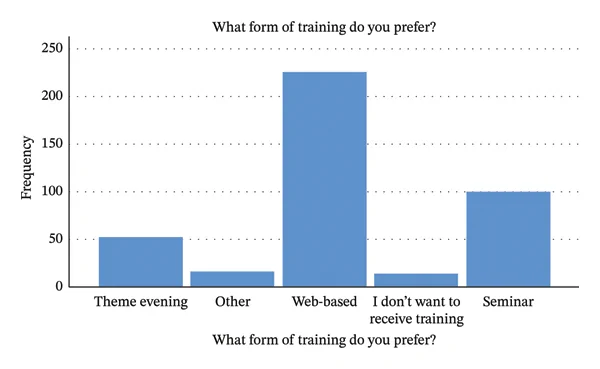
Preferred training modalities for pain management among pharmacists.

When asked about preferred formats for additional training, more than half of pharmacists indicated a preference for web‐based training, followed by seminars and themed evening sessions. Only a small minority reported no interest in further training. Training format preferences are illustrated in Figure 1.

**FIGURE 2:**
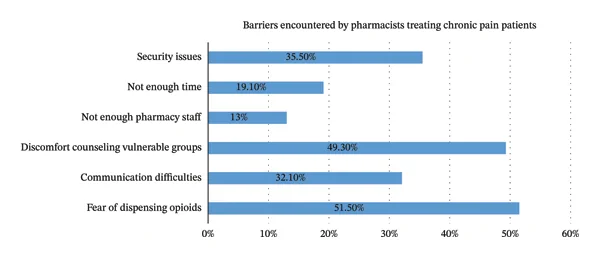
Barriers encountered by pharmacists in managing patients with chronic pain.

Figure 2 presents the distribution of barriers and challenges reported by pharmacists when managing patients with chronic pain. Fear of dispensing opioids was the most frequently reported barrier, identified by 51.5% of respondents. This was followed by discomfort when counseling vulnerable groups (49.3%). Other commonly reported challenges included communication difficulties, security concerns, and insufficient time. In contrast, inadequate pharmacy staffing was the least frequently reported barrier, reported by only 13% of participants.

Table 7 presents the results of the quantile regression analysis examining factors associated with pharmacists’ engagement in patient counseling practices. The knowledge score was a positive predictor of counseling behavior (*β* = 0.101, *p* < 0.001), indicating that higher assessed knowledge was associated with more frequent counseling. Self‐rated knowledge showed an even stronger association with counseling practices (*β* = 0.487, *p* < 0.001), suggesting that perceived competence plays an important role in pharmacists’ counseling behavior. In contrast, age, gender, educational level, years of experience, workplace setting, and region of practice were not significantly associated with counseling practices.

**TABLE 7:** Quantile regression analysis of factors associated with patient counseling practices.

Parameter estimates (*q* = 0.5)								
Parameter		Coefficient (*β*)	Std. error	*t*	df	Sig.	95% confidence interval	
							Lower bound	Upper bound
(Intercept)		8.877	1.3162	6.744	389	0.000	6.289	11.465

Age		0.004	0.0089	0.450	389	0.653	−0.014	0.022

Knowledge score		0.101	0.0245	4.103	389	0.000	0.052	0.149

Self‐reported knowledge of pain management (on a scale from 1 to 5)		0.487	0.1278	3.810	389	0.000	0.236	0.738

Gender	Females	0.105	0.2983	0.351	389	0.726	−0.482	0.691
	Males	0^c^	—	—	—	—	—	—

Educational level	Diploma	1.110E − 15	0.4814	0.000	389	1.000	−0.946	0.946
	Bachelor’s degree in Pharmacy	−0.491	0.3780	−1.299	389	0.195	−1.234	0.252
	Doctor of Pharmacy (Pharm.D)	−0.501	0.4562	−1.098	389	0.273	−1.398	0.396
	Postgraduate	0^c^	—	—	—	—	—	—

Years of experience	Recently graduated (< 1 year)	−0.755	0.4349	−1.735	389	0.084	−1.610	0.101
	1–4 years	0.028	0.4152	0.068	389	0.946	−0.788	0.844
	5–10 years	0.123	0.4649	0.264	389	0.792	−0.791	1.037
	> 10 years	0^c^	—	—	—	—	—	—

Workplace	Community pharmacy chain	−1.694E − 15	0.4360	0.000	389	1.000	−0.857	0.857
	Independent community pharmacy	0.292	0.4037	0.723	389	0.470	−0.502	1.085
	Governmental hospital	−0.408	0.5161	−0.791	389	0.429	−1.423	0.606
	Private hospital	0^c^	—	—	—	—	—	—

Work region	Southern region	−0.284	0.4121	−0.688	389	0.492	−1.094	0.527
	Northern region	−0.487	0.3061	−1.591	389	0.112	−1.089	0.115
	Central region	0^c^	—	—	—	—	—	—

*Note:* “c” is the reference group.

Table 8 presents the results of the ordinal regression analysis examining predictors of pharmacists’ self‐reported knowledge of laws governing controlled substances. Pharmacists holding a Doctor of Pharmacy (PharmD) degree had significantly lower odds of reporting higher knowledge levels compared with those with postgraduate qualifications (*β* = −0.848, *p* = 0.030). Lower levels of professional experience were also associated with reduced regulatory knowledge, with recently graduated pharmacists (*β* = −1.973, *p* < 0.001) and those with 1–4 years of experience (*β* = −1.500, *p* = 0.003) showing substantially lower odds of reporting higher knowledge compared with pharmacists with more than 10 years of practice. In addition, the absence of prior training in pain management was strongly associated with lower perceived knowledge of controlled‐substance laws (*β* = −0.750, *p* < 0.001). By contrast, age, gender, workplace setting, and region of practice were not significantly associated with knowledge levels.

**TABLE 8:** Ordinal regression analysis of pharmacists’ knowledge of controlled substance laws.

		Estimate	Std. error	Wald	df	Sig.	95% confidence interval	
							Lower bound	Upper bound
Age		−0.045	0.024	3.529	1	0.060	−0.092	0.002

Gender	Females	−0.388	0.253	2.349	1	0.125	−0.884	0.108
	Males	0^a^	—	—	0	—	—	—

Educational level	Diploma	−0.216	0.413	0.274	1	0.601	−1.025	0.593
	Bachelor’s degree in Pharmacy	−0.389	0.322	1.460	1	0.227	−1.020	0.242
	Doctor of Pharmacy (Pharm.D)	−0.848	0.391	4.714	1	0.030	−1.614	−0.083
	Postgraduate	0^a^	—	—	0	—	—	—

Years of experience	Recently graduated (< 1 Year)	−1.973	0.555	12.625	1	0.000	−3.062	−0.885
	1–4 Years	−1.500	0.500	8.991	1	0.003	−2.481	−0.520
	5–10 Years	−0.721	0.463	2.425	1	0.119	−1.629	0.187
	> 10 Years	0^a^	—	—	0	—	—	—

Workplace	Community pharmacy chain	0.568	0.374	2.306	1	0.129	−0.165	1.301
	Independent community pharmacy	−0.388	0.344	1.272	1	0.259	−1.063	0.286
	Governmental hospital	−0.190	0.449	0.179	1	0.673	−1.070	0.691
	Private hospital	0^a^	—	—	0	—	—	—

Work region	Southern region	−0.510	0.355	2.068	1	0.150	−1.206	0.185
	Northern region	−0.471	0.264	3.190	1	0.074	−0.988	0.046
	Central region	0^a^	—	—	0	—	—	—

Have you undergone training specifically related to pain management?	No	−0.750	0.206	13.240	1	0.000	−1.154	−0.346
	Yes	0^a^	—	—	0	—	—	—

*Note:* “a” is the reference group.

A quantile regression analysis was performed to identify factors associated with the overall pain management knowledge score. None of the examined variables showed a statistically significant relationship with the median knowledge score (Table 9).

**TABLE 9:** Quantile regression analysis of pharmacists’ pain management knowledge scores.

Parameter estimates (*q* = 0.5)								
Parameter		Coefficient	Std. error	*t*	df	Sig.	95% confidence interval	
							Lower bound	Upper bound
(Intercept)		38.619	1.7463	22.115	391	0.000	35.186	42.052

Age		0.012	0.0206	0.586	391	0.558	−0.028	0.053

Gender	Females	−0.715	0.6851	−1.044	391	0.297	−2.062	0.632
	Males	0^c^	—	—	—	—	—	—

Educational level	Diploma	−1.588	1.1099	−1.431	391	0.153	−3.770	0.594
	Bachelor’s degree in Pharmacy	−1.297	0.8710	−1.489	391	0.137	−3.009	0.415
	Doctor of Pharmacy (Pharm.D)	−1.315	1.0505	−1.252	391	0.211	−3.380	0.750
	Postgraduate	0^c^	—	—	—	—	—	—

Years of experience	Recently graduated (< 1 year)	0.115	0.9974	0.115	391	0.908	−1.846	2.076
	1–4 years	0.115	0.9542	0.120	391	0.904	−1.761	1.991
	5–10 years	0.382	1.0750	0.355	391	0.723	−1.732	2.495
	> 10 years	0^c^	—	—	—	—	—	—

Workplace	Community pharmacy chain	−0.012	1.0003	−0.012	391	0.990	−1.979	1.955
	Independent community pharmacy	−0.697	0.9212	−0.757	391	0.450	−2.508	1.114
	Governmental hospital	−1.073	1.1914	−0.900	391	0.369	−3.415	1.270
	Private hospital	0^c^	—	—	—	—	—	—

Work region	Southern region	−0.376	0.9526	−0.394	391	0.694	−2.249	1.497
	Northern region	0.321	0.7063	0.455	391	0.649	−1.067	1.710
	Central region	0^c^	—	—	—	—	—	—

*Note:* “c” is the reference group.

## 4. Discussion

This study examined the knowledge, attitudes, and practices of pharmacists working in both community pharmacies and hospital outpatient pharmacies in Jordan in relation to pain management, with particular attention to counseling behaviors, perceived competence, regulatory knowledge, and training needs. Overall, the findings suggest that pharmacists demonstrate generally good foundational knowledge and high levels of engagement in patient counseling, but with clear gaps in specific areas of clinical understanding, regulatory confidence, and formal training.

Pharmacists in this study demonstrated a strong understanding of several core principles of pain management, including the need to tailor analgesic choice to pain intensity, the use of combination analgesic therapy, and the role of nonpharmacological approaches such as CBT. This suggests that foundational elements of contemporary pain management are reasonably well established among pharmacists in this setting. Comparable patterns have been reported elsewhere, with community pharmacists showing generally good knowledge of core pain management concepts alongside more specific gaps [8, 14, 15]. In particular, studies from the Middle East similarly report strong endorsement of guideline‐consistent principles such as matching therapy to pain severity, multimodal strategies, and the value of nonpharmacological approaches while still identifying misconceptions that may affect care [14, 15]. Overall, these findings indicate that pharmacists in different settings tend to have a similar understanding of core pain management concepts, which may reflect shared training backgrounds and exposure to widely used guidance [25].

However, important knowledge gaps were also evident. A substantial proportion of pharmacists believed that infants experience less pain than adults, even though contemporary evidence shows that infants and children experience pain comparably to adults and may be especially vulnerable to adverse outcomes when pain is poorly managed; persisting assumptions of reduced pain perception in infants have been associated with a lower likelihood of adequate pain assessment and treatment, particularly outside specialist pediatric settings [26, 27].

Most pharmacists preferred web‐based training and placed high value on clinical guidelines, suggesting that flexible, guideline‐based continuing education would be well received. Previous studies have reported similar preferences among pharmacists for flexible, digitally delivered training, particularly for complex clinical areas such as pain management, where guidance evolves and confidence is closely linked to perceived competence [10, 15]. However, currently, there are no nationally developed clinical guidelines in Jordan specifically addressing pharmacists’ roles in pain management or providing detailed recommendations on analgesic selection and patient counseling. In practice, pharmacists rely on their professional training, clinical judgment, and international evidence when supporting patients with pain‐related concerns. Pharmacists also adhere to national dispensing regulations when supplying medications, particularly controlled substances [28]. The absence of a clear, locally oriented guideline may contribute to variability in clinical practices and highlights an opportunity to develop structured pain management guidance tailored to pharmacists’ scope of practice and the healthcare context in Jordan.

This need is further highlighted by observed misconceptions regarding the safety of commonly used analgesics. A notable proportion of pharmacists indicate that paracetamol is contraindicated in asthma and pneumonia. This contrasts with clinical evidence showing that paracetamol has an established safety profile and is widely recommended in guideline‐aligned practice, and with documented pharmacological understanding that NSAIDs carry specific ADR risks requiring contextual assessment [29–31]. Similar gaps in analgesic safety knowledge have been reported among community pharmacists in Saudi Arabia and the United Arab Emirates, suggesting that these issues are unlikely to be specific to the Jordanian context [14, 15]. This pattern points to limitations in how analgesic safety is taught and reinforced in practice, particularly with respect to translating pharmacological knowledge into condition‐specific counseling and decision‐making. These gaps partly reflect the structure and focus of undergraduate pharmacy education. Pharmacy programs in Jordan provide comprehensive training in pharmacology, therapeutics, and medication safety, including general principles of pain management. However, undergraduate education may place greater emphasis on theoretical knowledge rather than applied clinical decision‐making and patient‐centered counseling, particularly in complex areas such as pediatric pain assessment, chronic pain management, and opioid‐related risk communication. The observed gaps in specific areas of analgesic safety and pain perception are therefore broadly consistent with expectations based on university‐level training [32]. Together, these findings point to a critical need for enhanced education in pain management, not only at the undergraduate level but also for the systematic integration of pain management training programs and workshops into continuing professional development. In particular, incorporating pain management training within the mandatory continuing education hours required by the Jordan Pharmacists Association for practice license renewal may represent a strategic approach to strengthening pharmacists’ clinical contribution to pain management across healthcare settings.

Responses were mixed regarding whether chronic pain can be effectively managed using analgesics and adjuvant medications alone. This is an important finding, as it suggests that some pharmacists may continue to conceptualize chronic pain primarily through a biomedical lens, in which pharmacological treatment is viewed as sufficient in itself. Such perspectives contrast with a substantial body of evidence emphasizing the multidimensional nature of chronic pain and the importance of biopsychosocial approaches that integrate psychological, behavioral, and social interventions alongside medication [33, 34]. When pain is viewed primarily through a biomedical lens, engagement with nonpharmacological strategies may be more limited, potentially narrowing the scope of patient counseling.

At the same time, most pharmacists in this study recognized the role of antidepressants in improving symptoms and functional outcomes in people with chronic pain. This finding is consistent with evidence from systematic reviews and meta‐analyses demonstrating that certain antidepressant classes, particularly tricyclic antidepressants and serotonin–norepinephrine reuptake inhibitors, can provide modest but clinically meaningful benefits for pain and function across a range of chronic pain conditions, although effects vary by condition and drug class [35–37]. Overall, the findings suggest that while pharmacists are generally aware of the pharmacological management of chronic pain, nonpharmacological and psychosocial approaches may be less consistently embedded in how chronic pain is understood and managed.

Pharmacists reported high levels of engagement in patient counseling across all assessed domains, including education on side effects, drug interactions, therapeutic alternatives, and nonpharmacological strategies. The median counseling score was close to the maximum possible score, suggesting that counseling is a routine and embedded aspect of pharmacists’ practice in this setting. This finding is consistent with qualitative and quantitative studies highlighting the accessibility of community pharmacists and their frequent involvement in patient‐facing pain‐related consultations [9, 10].

Regression analyses showed that both objectively assessed knowledge and self‐rated knowledge were positively associated with the frequency of patient counseling, with perceived competence showing a stronger association than measured knowledge alone. This finding suggests that pharmacists’ confidence in their own abilities plays an important role in determining whether knowledge is translated into routine counseling practice. Evidence from recent pharmacy practice research indicates that pharmacists’ engagement in patient‐facing and extended clinical services is strongly influenced by perceived competence, role confidence, and readiness to act, rather than by demographic characteristics or practice setting alone [38, 39].

The absence of significant associations between counseling behavior and age, years of experience, or workplace setting in this study further supports the importance of individual‐level factors over structural characteristics. Similar findings have been reported in studies examining pharmacists’ involvement in clinical and advisory roles, where confidence and perceived preparedness were more predictive of service delivery than professional seniority or environment [38]. Taken together, these results suggest that initiatives aimed at strengthening pharmacists’ contribution to pain management may be most effective when they focus on building applied confidence and counseling skills, alongside clinical knowledge.

While pharmacists generally rated their education and regulatory knowledge as moderate, the regression analysis revealed that pharmacists with lower levels of experience and those without prior pain management training were significantly less likely to report higher knowledge of controlled substance regulations. Interestingly, pharmacists holding a PharmD degree reported lower perceived regulatory knowledge compared with those with postgraduate qualifications, which may reflect curricular variation or differences in exposure to regulatory frameworks during training.

Fear of dispensing opioids emerged as the most frequently reported barrier to managing patients with chronic pain, followed closely by discomfort when counseling vulnerable groups. This finding is consistent with international evidence showing that community pharmacists often experience uncertainty and anxiety around opioid‐related decision‐making, driven by concerns about regulatory scrutiny, legal liability, misuse, and stigma associated with opioid prescribing and dispensing [10, 11, 13]. Such concerns have been shown to limit pharmacists’ willingness to engage fully in pain management discussions, particularly in complex or high‐risk cases, despite their accessibility and clinical expertise.

Notably, insufficient staffing was the least frequently reported barrier in this study. This suggests that structural constraints alone do not explain reduced engagement in pain management and that psychological and professional factors, including confidence, regulatory clarity, and perceived role legitimacy, may play a more influential role. Similar conclusions have been drawn in previous research, where pharmacists’ involvement in pain management was shaped less by workload and more by training, confidence, and clarity around professional responsibilities [10, 13].

## 5. Implications for Practice

These findings have clear implications for community pharmacy practice and professional development. While pharmacists showed high levels of engagement in patient counseling and generally good foundational knowledge, gaps in specific areas of pain management point to the need for training that goes beyond broad pharmacological principles. In particular, misconceptions around pediatric pain, analgesic safety, and nonpharmacological approaches suggest value in training that focuses more on clinical judgment and real‐world decision‐making.

The strong link between perceived competence and counseling frequency suggests that confidence plays an important role in shaping practice. Educational initiatives may therefore need to go beyond providing clinical information and focus on helping pharmacists feel more confident applying this knowledge in patient‐facing situations, particularly when discussions are complex or sensitive, such as those involving chronic pain or opioids. Training that includes practical examples, clear regulatory guidance, and realistic counseling scenarios may be especially helpful.

Fear of dispensing opioids was a prominent barrier, which points to the need for clearer regulatory guidance and more targeted education around controlled substances. Reducing uncertainty about legal and professional responsibilities may help ease anxiety and support more consistent engagement with people living with chronic pain. Given pharmacists’ preference for web‐based training and the importance they place on clinical guidelines, online, guideline‐based education may offer a practical way to support ongoing professional development in this area.

### 5.1. Strengths, Limitations, and Future Directions

This study has several strengths. It provides one of the first comprehensive examinations of community pharmacists’ knowledge, attitudes, practices, and perceived barriers related to pain management in Jordan. The inclusion of both self‐reported measures and objectively assessed knowledge, alongside regression analyses, allowed for a more nuanced understanding of the factors associated with counseling behavior. The relatively large sample size and inclusion of pharmacists from different practice settings and regions further strengthen the relevance of the findings.

Several limitations should also be acknowledged. The cross‐sectional design precludes causal inference, and the reliance on self‐reported data introduces the potential for social desirability and recall bias, particularly in relation to counseling behaviors and perceived competence. The study sample was predominantly young and early in career, which may limit the generalizability of findings to more experienced pharmacists. In addition, while the questionnaire was adapted from a validated instrument and underwent careful translation and piloting, some constructs, such as counseling quality and depth, could not be captured in detail. In addition, because the survey was distributed through a combination of in‐person recruitment and online professional networks, the total number of eligible pharmacists who received or had access to the survey could not be determined. As a result, a response rate could not be calculated, and the representativeness of the sample should therefore be interpreted with caution.

Future research could build on these findings in several ways. Qualitative studies may help explore pharmacists’ experiences of pain management, opioid‐related decision‐making, and regulatory uncertainty in greater depth. Longitudinal or intervention studies could examine whether targeted, guideline‐based training improves both confidence and clinical practice over time. Finally, exploring patient perspectives on pharmacist‐delivered pain counseling would provide valuable insight into how pharmacists’ roles in pain management are perceived and where further improvements may be needed.

## 6. Conclusion

This study shows that community pharmacists in Jordan are actively involved in pain management and routinely provide patient counseling, supported by generally good foundational knowledge. However, gaps remain in specific areas, including pediatric pain, analgesic safety, nonpharmacological approaches, and confidence around opioid‐related decision‐making. These findings point to the value of training that supports applied clinical judgment and regulatory clarity, rather than knowledge acquisition alone, to strengthen pharmacists’ role in chronic pain care.

## Funding

This research received no specific grant from any funding agency in the public, commercial, or not‐for‐profit sectors.

## Conflicts of Interest

The authors declare no conflicts of interest.

## Supporting Information

Additional supporting information can be found online in the Supporting Information section.

## Supporting information


**Supporting Information** Appendix Table A1. National versus sample distribution of community pharmacists by region.

## Data Availability

The data that support the findings of this study are available from the corresponding author upon reasonable request.
