# Supplementary material for: Pain Management in Patient‐Facing Pharmacy Settings in Jordan: Pharmacists’ Knowledge, Attitudes, and Practices
Source: Pain Res Manag. 2026 Aug 28;2026:9198210. doi: 10.1155/prm/9198210 (PMC13522928; doi:10.1155/prm/9198210)
Supplement: Supplementary file 1 [file PRM-2026-9198210-s001.docx]

Table A1. National vs sample distribution of community pharmacists by region, n (%)

| Region | Total | Sample |
| --- | --- | --- |
| Northern | 855 (23.8%) | 69 (16.9%) |
| Southern | 307 (8.5%) | 34 (8.3%) |
| Central | 2423 (67.6%) | 305 (74.8%) |

Note. Total reflects national workforce counts of registered community pharmacists.
